# VEGF-Loaded Heparinised Gelatine-Hydroxyapatite-Tricalcium Phosphate Scaffold Accelerates Bone Regeneration *via* Enhancing Osteogenesis-Angiogenesis Coupling

**DOI:** 10.3389/fbioe.2022.915181

**Published:** 2022-06-08

**Authors:** Xu Chen, Chun-Yan Gao, Xiao-Yang Chu, Chun-Yan Zheng, Ying-Yi Luan, Xin He, Kai Yang, Dong-Liang Zhang

**Affiliations:** ^1^ Department of Orthodontics, Beijing Stomatological Hospital, Capital Medical University School of Stomatology, Capital Medical University, Beijing, China; ^2^ Department of Stomatology, Eighth Medical Center of Chinese PLA General Hospital, Beijing, China; ^3^ Department of Stomatology, Fifth Medical Center of Chinese PLA General Hospital, Beijing, China; ^4^ Prenatal Diagnosis Center, Beijing Obstetrics and Gynecology Hospital, Capital Medical University, Beijing, China

**Keywords:** heparin, VEGF, gelatine, hydroxyapatite, tricalcium phosphate, bone regeneration

## Abstract

**Background:** Bone tissue defect, one of the common orthopaedicdiseases, is traumatizing and affects patient’s lifestyle. Although autologous and xenograft bone transplantations are performed in bone tissue engineering, clinical development of bone transplantation is limited because ofvarious factors, such as varying degrees of immune rejection, lack of bone sources, and secondary damage to bone harvesting.

**Methods:** We synthesised a heparinised gelatine-hydroxyapatite-tricalcium phosphate (HG-HA-TCP) scaffold loaded with sustained-release vascular endothelial growth factor (VEGF) analysed their structure, mechanical properties, and biocompatibility. Additionally, the effects of HG-HA-TCP (VEGF) scaffolds on osteogenic differentiation and vascularisation of stem cells from human exfoliated deciduous teeth (SHED) *in vitro* and bone regeneration *in vivo* were investigated.

**Results:** HG-HA-TCP scaffold possessed good pore structure, mechanical properties, and biocompatibility. HG-HA-TCP scaffold loaded with VEGF could effectively promote SHED proliferation, migration, and adhesion. Moreover, HG-HA-TCP (VEGF) scaffold increased the expression of osteogenesis- and angiogenesis-related genes and promoted osteogenic differentiation and vascularisation in cells. *In vivo* results demonstrated that VEGF-loaded HG-HA-TCP scaffold improved new bone regeneration and enhanced bone mineral density, revealed byhistological, micro-CT and histochemical straining analyses. Osteogenic and angiogenic abilities of the three biological scaffolds wereranked as follows: HG-HA-TCP (VEGF) > G-HA-TCP (VEGF) > G-HA-TCP.

**Conclusion:** HG-HA-TCP (VEGF) scaffold with good biocompatibility could create an encouraging osteogenic microenvironment that could accelerate vessel formation and osteogenesis, providing an effective scaffold for bone tissue engineering and developing new clinical treatment strategies for bone tissue defects.

## Highlights

• HG-HA-TCP scaffold had good pore structure, mechanical properties, and biocompatibility.
• VEGF-loaded HG-HA-TCP scaffold could effectively promote SHED proliferation, migration, and adhesion.
• VEGF-loaded HG-HA-TCP scaffold could effectively promote osteogenic differentiation and vascularisation.
• VEGF-loaded HG-HA-TCP scaffold improved new bone regeneration and enhanced bone mineral density.

## Introduction

Bone tissue defect, a common orthopaedic disease, is primarilycaused by trauma, developmental deformity, revision surgery, osteomyelitis. It causesgreat pain (Zhu et al., 2022). The most common solution for bone defect restoration is to gain bone mass through transplantation of bone tissue engineering scaffolds, which are commonly considered autologous bones and xenografts (Zizzari et al., 2016). However, an autologous bone graft is limited in source, prone to infection in the donor site, and involvescomplicated surgical procedures, causing considerable limitations to its development. At this stage, the application of autologous bone grafts cannot provide satisfactory results for bone defect repair (Fillingham and Jacobs, 2016).

On the other hand, an allogeneic bone graft has structural properties similar to autologous bone and is easy to obtain and process, yet it risks immune rejection and infection (Wu et al., 2021). Therefore, researchand development of alternative pathways for bone defect repair have been a hotspot for research. Bone tissue engineering emerged, and synthetic biomaterials have also been widely studied and applied to solve the problem of bone defect repair (Wang et al., 2019).

Bone tissue engineering is a discipline that couples cell biology with material science to construct tissues or organs *in vitro* and *in vivo*, with three elements of seed cells, biomaterials, and biological factors (Marolt et al., 2010; Roddy et al., 2018). An optimal bone biomaterial should possess satisfactory mechanical properties, excellent biocompatibility and degrade at a moderate rate to match bone regeneration, which is crucial to bone defect repair (Mistry and Mikos, 2005). All artificial bone materials have evolved and refined, including glass ceramics, metals, and organic polymers (Dorozhkin, 2010). Since it is difficult for a single scaffold material to meet the material requirement of bone tissue engineering, two or more scaffold materials are often used after compounding (Gu et al., 2022). Tricalcium phosphate (TCP) and hydroxyapatite (HA) are important components of human bones and are extensively studied in artificial bone materials (Konovalenko et al., 2021). Gelatine (G) is a soluble collagen substitute with the advantages of low antigenicity, easy use, and low price; it is widely used in bone tissue engineering (Salehi et al., 2018). A previous study indicated that HA crystals could increase the strength of collagen, which could also enhance the toughness of HA crystals (Wolters et al., 2017). In our previous study, a G-HA-TCP composite scaffold was constructed, and it was confirmed by *in vitro* experiments that it could induce fine osteogenesis of human dental pulp stem cells (SHED) (Gu et al., 2018).

Bone tissue is highly vascularised; itsrenewal involvesinteraction between osteogenesis and angiogenesis, further leading to bone formation and tissue repair (Clark et al., 2015). Bone regeneration depends on successful angiogenesis, which is highly related toselecting tissue-engineered grafts (Novosel et al., 2011; Diomede et al., 2020). An optimal scaffold should ensure the development of a vascular network, providing a positive and appropriate microenvironment fortissue engineering and renewal (Martino et al., 2015). A wide range of growth factors can promote the vascularisation of seed cells and bone tissue material complexes. Among them, vascular endothelial growth factor (VEGF), also an angiogenesis factor, has the most potent ability to rebuild blood vessels and is often used in combination with scaffolds to improve osteogenic efficiency (Zha et al., 2021). *In vivo*, VEGF can be reserved in the natural extracellular matrix by its interactionwith sulphated glycosaminoglycans (such as heparin or heparan sulphate), and many studies mimicked the *in vivo* situation by complexing the scaffold with heparin to maintain the biological activity of VEGF while controlling itsrelease (Claassen et al., 2017).

However, whether VEGF-loaded heparinised G-HA-TCP (HG-HA-TCP) scaffold could regulate osteogenesis-angiogenesis coupling and bone regeneration has not been studied before. Previous research of our group found that G-HA-TCP could induce the osteogenic differentiation of SHED *in vitro*, which laid the foundation for present research. In the current study, we fabricated an HG-HA-TCP scaffold, and the physical properties, biocompatibility, and osteogenic properties after loading with VEGF were further observed and studied.

## Materials and Methods

**FIGURE 1:**
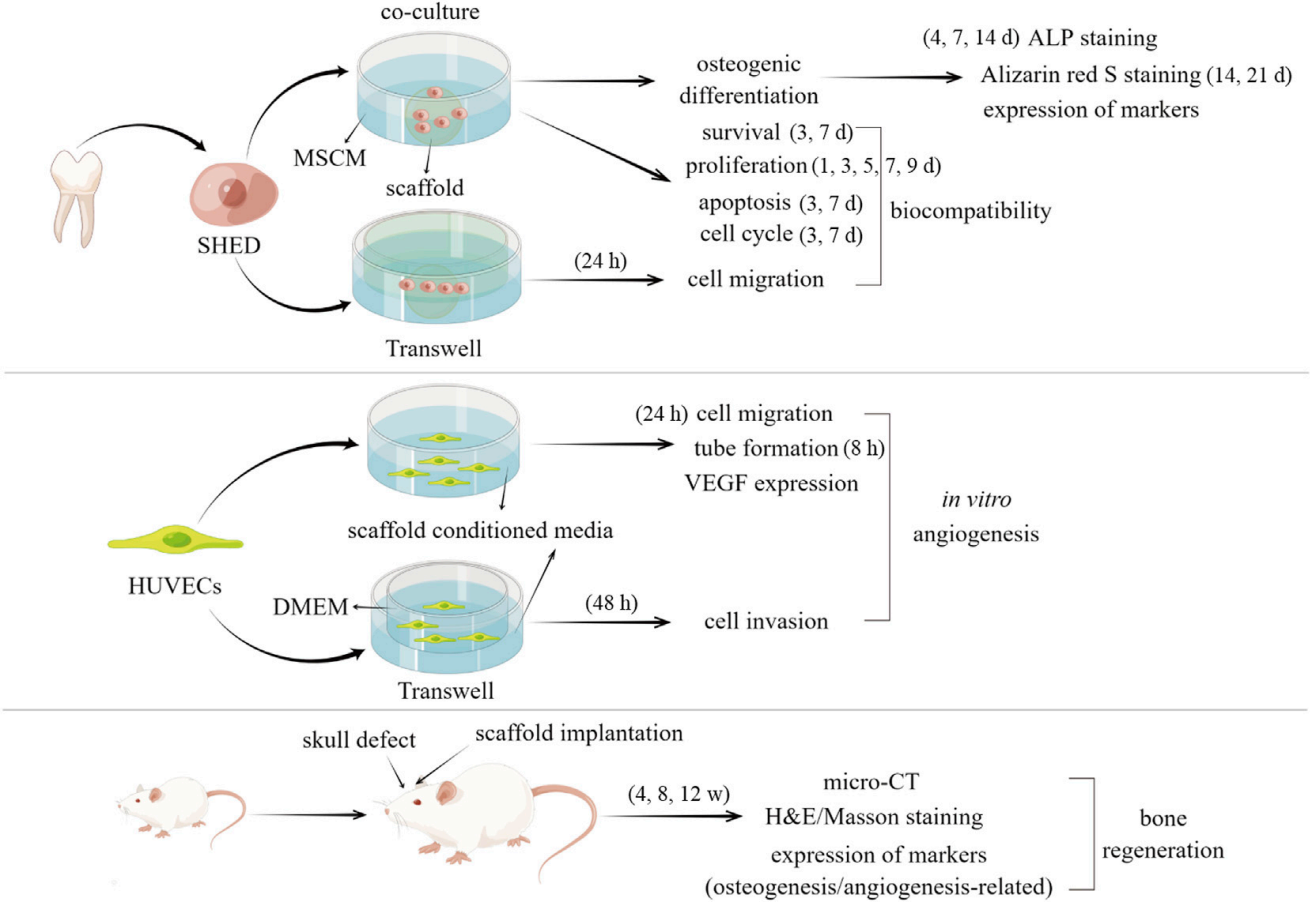
Diagram of the whole process of this study.

The schematic diagram of whole process of the experiments was demonstrated in Figure 1.

### Synthesis of G-HA-TCP Scaffold

The mechanical strength of HA is similar to that of normal bone tissue, but it has the disadvantages of poor mechanical properties, high brittleness, easy fracture, and refractory degradation. TCPbioceramics are similar to natural inorganic bone components with good osteoconductivity, osteoinductivity and biocompatibility, which are non-toxic and can be degraded and absorbed. However, it has the disadvantages of fast degradation, poor plasticity and unstable mechanical properties. Therefore, HA and TCP are often used in combination to complement each other for better performance of the stent. G has the characteristics of low strength, high brittleness, easy water absorption and swelling, and extremely low strength and elastic modulus after swelling. The properties of gelatin porous scaffolds are usually improved by physical modification and chemical cross-linking. G-HA-TCP was synthesised as per a previous study (Gu et al., 2018). In brief, G, HA, and TCP (at a proportion of 10:1:1) were added to ultrapure water and sonicated for 20 min, followed by a hot-water bath for 1 h. Sodium chloride was added as a pore-forming agent in the mixtureand simultaneously mixed with glutaraldehyde as a cross-linking agent. After incubating for 30 min, the formed scaffold was sodden and cleaned with ultrapure water for 12 h and aged 24 h under room temperature to obtain a gel-like 3D porous G-HA-TCP scaffold.

### Conjugation of Heparin Onto G-HA-TCP Scaffold

G-HA-TCP scaffold was submerged in MES buffer for 2 h to prepare scaffold solution. Besides, N-Hydroxysuccinimide (NHS) and 1-(3-Dimethylaminopropyl)-3-ethylcarbodiimide hydrochloride (EDC) were added tothe heparin solution and incubated for 15 min to stimulate heparin (with a molar proportion of EDC:NHS as 1:1). Subsequently, scaffold solution was mixed into heparin solution with gentle stirring for 24 h atroom temperature. Finally, distilled water was used to rinse the scaffold to remove sideproducts. After freeze-drying, HG-HA-TCP material was obtained.

### Microstructural Characterisation of Scaffolds

Freeze-dried G-HA-TCP and HG-HA-TCP scaffolds were fixed on the conductive tape on the sample holder, which was furtherplaced in the gold steaming chamber and gold was sprayed when it reached a vacuum state. Carl Zeiss scanning electron microscope (SEM) was used to observe surface morphology, pore distribution, and pore size of scaffolds under an energy dispersive spectrum voltage of 3 kV.

### Preparation of Scaffolds Loaded With VEGF

VEGF-loaded scaffolds were prepared by the adsorption method. Briefly, 2 μg of recombinant human VEGF165 (127464-60-2, Peprotech, Rocky Hill, NJ, United States) was dissolved in 2 ml of ultrapure water consisting of 0.1% bovine serum albumin (BSA). Further, ethylene-oxide-sterilised HG-HA-TCP and G-HA-TCP scaffolds were placedin VEGF solution and keptat 4°C for 18 h. After washing, VEGF loaded scaffolds were dried under vacuum conditions and stored under room temperature.

### Isolation and Identification of SHED

Twenty children with retained deciduous teeth treated in the Department of Paediatric Stomatology of our hospital were selected. With the notification and consent of their guardians, extracted deciduous teeth were obtained. The current study obtained approval fromthe Ethics Committee of Capital Medical University, Beijing, China (No. CMUSH-IRB-KJ-PJ-2020-02). The surface of the tooth was sanitised with 75% ethanol; the pulp was detached under hygienic conditions, cut into asizeof 1–1.5 mm^3^, and digestedwith 0.4% neutral protease and 0.3% type I collagenase for 30 min at 37°C. MSCM medium (HyClone, United States) containing 20% foetal bovine serum (FBS; Gibco, United States) was addedto cease theenzymatic activity. After the digestion was terminated, cells were transferred to a 60-mm Petri dish with an MSCM medium supplemented with 100 U/mL streptomycin (Gibco), 100 U/mL penicillin (Gibco), and 15% FBS in 10% CO_2_ at 37°C for conventional culture, and the medium was changed every 2–3 days.

Flow cytometry was performed to detect surface antigens to analyse the expression of specific mesenchymal protein markers. The third-generation SHED was harvested, 1 × 10^6^ cells were resuspended in50 μl cold PBS. Further, cells were tagged with phycoerythrin (PE)-conjugated antibodies against CD105 (4300023, eBioscience, San Diego, CA, United States) and CD34 (119307, Biolegend, San Diego, CA, United States) and fluorescein isothiocyanate (FITC)-conjugated antibodies targeting CD44 (103,005, Biolegend), CD90 (328107, Biolegend), CD45 (304005, Biolegend), CD14 (301803, Biolegend), and CD11b (4271325, eBioscience). After 30 min of incubation, cells were rinsed with cold PBS solution and placed in BD Accuri™ C6 Plus flow cytometer (BD Biosciences, Bedford, MA, United States) for detection. Data were analysed using Cell Quest software (BD Biosciences). For the detection of cell differentiation, cells were treated with osteogenic (HUXXC-90021) and adipogenic (HUXXC-90031) induction medium (Cyagen, Guangzhou, China) for 2–3 weeks. Further, cells were assessed with Alizarin red S and Oil red O staining.

### Co-Culture of Cells and Scaffolds

Third-generation SHED wasdigested with 0.25% trypsin, and MSCM medium was added to make a cell suspension. Ethylene-oxide-sterilised scaffold material was placed in the third-generation cell suspension containing 1 × 10^5^ cells, then shookfor 1 h at 37°C. Further, thescaffold materials were taken out and placed in a petri dish, onto which the remaining cell suspension was dripped. After 4 h, it was cultured in an MSCM medium.

### Cell Proliferation Assay

Cell proliferation assay was conducted with CCK-8 kits (CK04, DoJinDo, Japan). Briefly, cell suspension (100 μl/well) was seeded in a 96-well plate, and the cells were co-cultured with the scaffold after pre-incubationof 24 h (37°C, 5% CO_2_). On days 1, 3, 5, 7, and 9 of co-culture of cells and scaffolds, 10 μl of CCK-8 solution was added to the cells. After incubation for 4 h, the absorbance of each well was measured at 450 nm usinga microplate reader.

### Live/Dead Cell Staining

Cells on scaffolds on days 3 and 7 of culture were stained with LIVE DEAD Viability Cytotoxicity Kit (1976809, Invitrogen, Carlsbad, CA, United States) for cell survival imaging assay. Briefly, 0.5 μl Calcein AM (stains live cells, glows green) and 2 μl Eth DB (stains dead cells, glows red) were added to 1 ml PBS solution to prepare a working staining solution. Cells were cultured for 15–20 min at room temperature in the dark and observed usinga fluorescence microscope as soon as possible after the incubation. The proportion of living and dead cells wascounted, and the cells were photographed.

### Migration and Invasion Assays

Invasive (HUVECs, from ATCC) and migratory (SHED) abilities of cells were evaluated usingMatrigel-coated inserts (BD Biosciences) and Transwell assay inserts (8 μm PET, 24-well Millicell), respectively. Overall, 200 μl MSCM or DMEM containing 2 × 10^5^ cells and 800 μl medium comprising 10% FBS and various scaffolds were added to the upper and lower compartments. The cells were cultured at 37°C with 5% CO_2_ for 48 h (invasion) or 24 h (migration). The invaded or migrated cells of the lower chambers were rinsed 3 times with PBS and fixed with paraformaldehyde for 10 min. After rinsing and air-drying, the cells were stained with 0.1% crystal violet for 10 min and observed usinga microscope.

### Cell Cycle Detection

A cell cycle detection kit was purchased from Bioss (BA00204, Beijing, China). After co-cultivation with scaffolds for 3 or 7 days, supernatant liquid of cells was collected, cells were trypsinised, and MSCM was added. After washing cells twice with PBS, 70% ethanol was added to the cells. Cells were fixed at 4°C for 24 h and centrifuged (2000 rpm, 5 min), then removedthe supernatant. PBS was added to the cells, and the suspension was centrifuged (2000 rpm, 5 min) again, followed by the removal of the supernatant. The cells wereresuspended in 100 μl RNase A and placed in a water bath at 37°C for 30 min. Further, 400 μl PI was added to the cells, incubated at 4°C for 30 min in the dark. The cell cycle was examined using flow cytometry.

### Apoptosis Analysis

The apoptosis kit was purchased from Nanjing KGI Biotechnology (KGA108-1, Nanjing, China). After co-cultivation of cells and scaffolds for 3 or 7 days, cells were harvested by trypsinisation without EDTA, rinsed twice with PBS, and resuspended in 500 μl of binding buffer. Further, the cells were incubated with 10 μl Annexin V-FITC in the dark for 10 min atroom temperature. Additionally, 5 μl of PI was added to the cells, and they were incubatedfor another 5 min under the same experimental conditions. Cell apoptosis was examined usingflow cytometry.

### Analysis of Alkaline Phosphatase Activity

SHED wasseeded into three groups of scaffold materials in an osteogenic medium (HUXXC-90021, Cyagen) at a seeding density of 1 × 10^4^ cells/well. After 4, 7, and 14 days of culture, the culture mediumwas removed, and ALP activity was measured per the manufacturer’s instructions withthe alkaline phosphatase detection kit.

### Alizarin Red S Staining

Alizarin red S staining solution (ALIR-10001, Cyagen) was used in the present procedure. Overall, 1 × 10^4^ cells/well were seeded into 6-well plates and incubated with osteogenic medium at37°C with 5% CO_2_ for 24 h. The initial medium was replaced every 2 days after incubation. After 14 and 21 days of incubation, cells were rinsed twice with PBS andfixed with 4% paraformaldehyde for 30 min. After PBS rinsing 2–3 times, cells were stained with 2 ml Alizarin red S staining solution for 10 min and decolourised. Further, 2 ml PBS was added to each well. After 15 min of incubation, absorbance was measured at 490 nm using a microplate reader.

### Quantitative Real-Time Polymerase Chain Reaction

Third-generation SHED wasseeded in 6-well plates at a density of 1 × 10^4^ cells/well and co-cultured with osteogenic medium and scaffolds for 14 and 21 days to obtain cell pellets. Total RNA was extracted with TRIzol reagent (Invitrogen, Carlsbad, CA, United States), and cDNA was synthesised using PrimeScript RT Master Mix Perfect Real-Time Kit (Takara, Nanjing, China). SYBR Select MasterMix (Takara) was used for qRT-PCR assay to detect the expression of osteogenic differentiation markers, including ALP, bone salivary protein (BSP), runt-related transcription factor 2 (RUNX2), osterix (OSX), osteocalcin (OCN), and VEGF (in SHED and HUVECs). Glyceraldehyde-3-phosphate dehydrogenase (GAPDH) was usedas an internal reference gene. All primers used in the current study was purchased from Genechem (Shanghai, China).

### Western Blotting

Osteogenic medium, scaffolds, and seeded cells were collected and co-cultured for 21 days to obtain cells. RIPA lysis buffer (Beyotime Biotechnology, Shanghai, China) was added to extract total cell proteins. Protein concentration was detected usingthe BAC method (Beyotime Biotechnology), and a 4 μg/μl protein solution was prepared. The proteinswereseparated by 12% SDS-PAGE electrophoresis and transferred to a PVDF membrane (Millipore, Billerica, MA, United States). After blockingwith 5% skimmed milk for 1 h, membranes were cultured with primary antibodies as follows: ALP (1:1000, ab229126, Abcam, Cambridge, United Kingdom), BSP (1:1000, #5468, Cell Signaling Technology), OCN (1:1000, ab133612, Abcam), OSX (1:1000, ab209484, Abcam), RUNX2 (1:1000, ab236639, Abcam), OCT4 (1:1000, ab181557, Abcam), SOX2 (1:1000, ab92494, Abcam), Nanog (1:1000, ab109250, Abcam), VEGF (1:1000, bs-0279R, Bioss), and GAPDH (1:2500, ab9485, Abcam). The next day, secondary antibodies (A0208, Beyotime Biotechnology) were added to the membrane, and the protein bands were detected usingECL (NCI5079, Thermo Fisher Scientific, Shanghai, China).

### Wound Healing Assay

Different scaffold solutions were prepared before the wound healing assay by adding DMEM + 1% BSA with different scaffolds for 24 h at a scaffold surface area to a media volume proportion of 1.25 cm^2^/ml. To prepare scaffold/monocyte conditioned media, monocytes were incubated in scaffold solutions for 24 h. HUVEC cells were inoculated in 24-well cell culture plates. DMEM medium was discarded when cell confluence reached 90%, and serum-free medium was added for 24 h. When cell confluence reached 100%, cells were scratched using apipette tip, and a corresponding conditioned medium from various scaffolds was added. Cell scratches were photographed using an inverted microscope. The scratch area S at 0 (S0) and 24 h (S24) wereanalysed using ImageJ software. The cell migration rate was calculated as follows:

Cell migration rate =(S0-S24)/S24×100%

### Matrigel Tubule Formation Experiment

The effects of scaffolds on the formation of the vascular tube by HUVEC cells were evaluated usingan *in vitro* angiogenesis assay kit (Corning). Cells (1.5 × 10^4^ cells/well) were seeded in a Matrigel-coated 96-well plate and incubated for 8 h in various conditioned media. An inverted microscope was used for observing the formation of tubular structures in endothelial cells, and three areas of view in each well were selected randomly for photographing.

### *In vivo* Implantation

Thirty-six male SD rats (Beijing Charles River Laboratories) weighing 250–300 g was placed in a clean and sterile environment with an ambient temperature of 22 ± 1°C and a day-night cycle of 12 h. They were divided randomly into control, G-HA-TCP scaffold, G-HA-TCP (VEGF), and HG-HA-TCP (VEGF) groups. Rats were anaesthetized by intraperitoneal injection of sodium pentobarbital (30 mg/kg). The periosteum was detached from the bone surface, drilled to create a circular skull defect with a diameter of 5 mm and implanted with various scaffolds. After implantation, skull tissue was collected for follow-up evaluation after 4, 8, and 12 weeks of implantation. For animal experiments, approval was obtained from the Ethics Committee of Capital Medical University (Beijing, China; MDKN-2021–046).

### Micro-CT Analysis

SD rats in each group (*n* = 6) were anaesthetized using3% pentobarbital sodium. Further, a micro-CT system was used to calculate the bone mineral density (BMD) and bone volume/total volume (BV/TV) fraction of regenerated bone in the calvarial defect. The micro-CT device was set with a tomographic rotation of 180° at 85 kV and 135 mA.

### Histology Evaluation

Rats were sacrificed at 4, 8, and 12 weeks after implantation, and the scaffold and surrounding tissues were collected. The sample was soaked in 10% formalin for 48 h, and ethylenediaminetetraacetic acid (EDTA) solution (pH = 8.0, 0.5 M) was added for decalcification for 30 days. The decalcified samples were embedded in paraffin and sliced into thick sections of 4 μm for histological observation. To assess the regeneration of skull defect, slices were stained with Masson’s trichrome and haematoxylin and eosin (H&E). In the current study, the 3Dhistech Pannoramic Scan slide scanning system was used to scan the bone tissue sections after H&E and Masson staining. Case Viewer software was used for observation and analysis.

### Immunohistochemical Analysis

For immunohistochemistry, tissue sections were blocked with 5% BSA for 30 min and cultured with primaryanti-BMP2 (1:200, PA5-85956, Thermo Fisher), anti-CD31 (1:50, PA5-16301, Thermo Fisher), anti-RUNX2 (1:200, PA5-82787, Thermo Fisher), and anti-vWF (1:100, PA5-16634, Thermo Fisher). The optical density of immunohistochemical staining results was quantitatively evaluated usingImage J software.

### Data Analysis

Measurement data were expressed as mean ± standard deviation (SD). Comparison between experimental groups wasconducted usinga one-way analysis of variance, followed by Tukey’s multiple comparisons tests using SPSS 19.0 (SPSS Inc., Chicago, IL, United States) or GraphPad Prism seven software (GraphPad Software, La Jolla, CA, United States). *p* < 0.05 was consideredsignificant.

## Results

### Characteristics of Scaffolds

**FIGURE 2:**
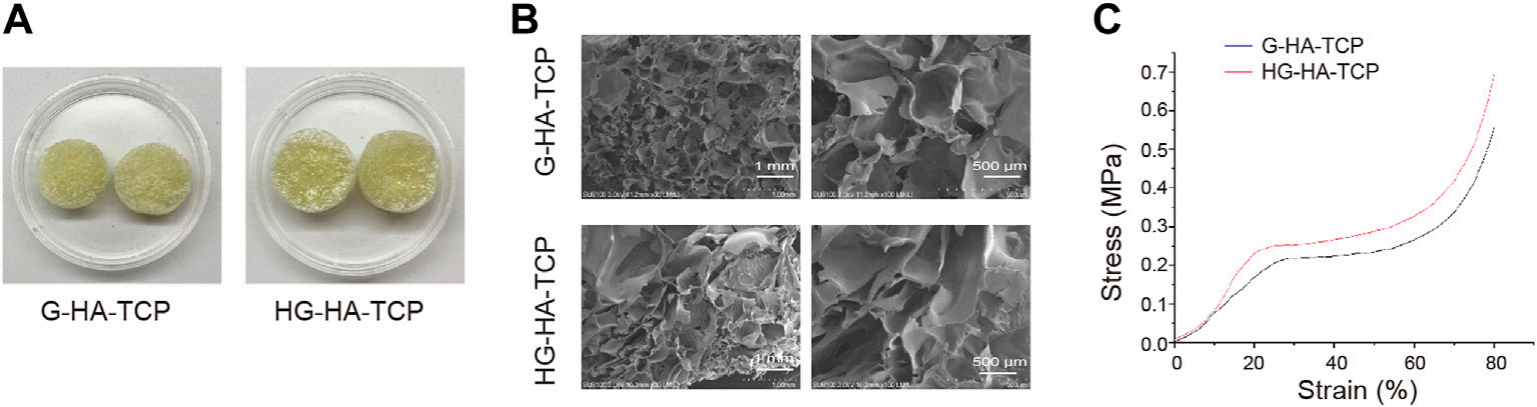
Characteristics of scaffolds. **(A)** Surface morphology of scaffolds. **(B)** Internal structure of scaffolds as observed usingSEM. **(C)** Stress-strain curve of scaffolds.

First, we observed the characteristics of HG-HA-TCP and G-HA-TCPscaffolds. Both scaffolds were faint yellow incolour with loose and porous surfaces, and HG-HA-TCP hada brighter gloss than G-HA-TCP (Figure 2A). Scanning electron microscopy revealed that the pore size of the G-HA-TCP scaffold ranged from 70–200 μm with large porosity. HG-HA-TCP scaffold had alternating pore sizes, and the pore size range was 50–200 μm. Besides, clear pore structure could be seen in scaffolds, with large porosity, rich pores, and uniform distribution (Figure 2B). Results of the stress-strain curve of the scaffolds revealed that it was smooth when the deformation was 0%–20%. When the stress reached 0.2 MPa, the G-HA-TCP scaffold was yielded, and when the stress reached 0.25 MPa, the HG-HA-TCP scaffold was yielded. However, no notable difference was observed in the elastic moduli of the two scaffolds (Figure 2C). The results revealed that prepared HG-HA-TCP scaffolds were partially modified based on G-HA-TCP scaffolds, and the mechanical properties did not change markedly. With suitable pore size and wide pore size distribution, scaffold materials could meet the requirements of angiogenesis and cell ingrowth and wereoperational during bone tissue engineering.

### Isolation and Identification of SHED

**FIGURE 3:**
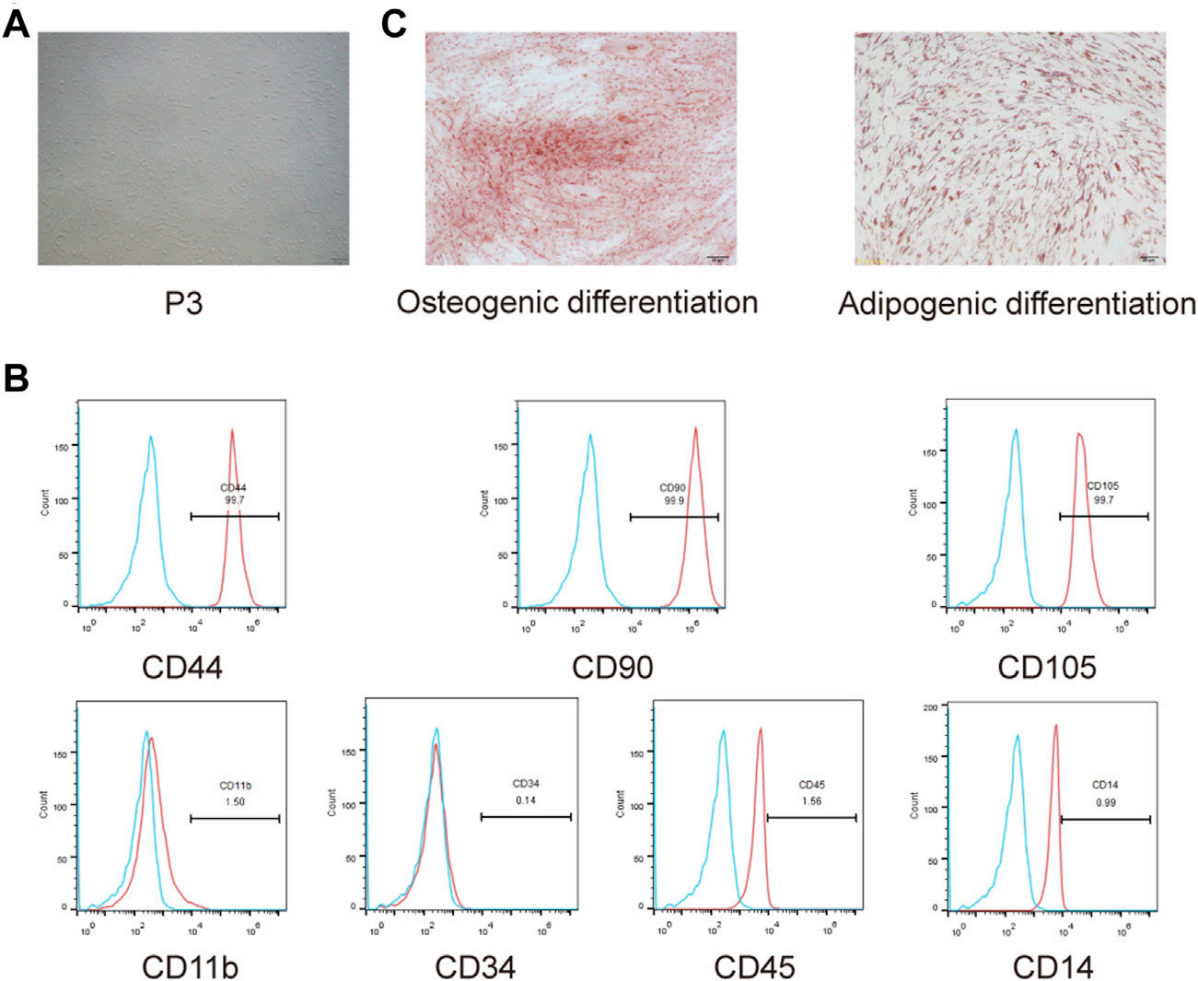
Isolation and identification of SHED. **(A)** Morphology observation of isolated cells from exfoliated deciduous teeth. **(B)** CD44, CD90, CD105, CD11b, CD34, CD45, and CD14 expressions were measured usingflow cytometry. **(C)** Osteogenic andadipogenic differentiation abilities of isolated cells were assessed.

The isolated cells had a typical fibroblast-like morphology (Figure 3A). Flow cytometricanalysis revealed that the cells expressed the mesenchymal stem cell markers CD44, CD90, and CD105, whereas the hematopoietic cell markers CD11b, CD34, CD45, and CD14 were negatively expressed (Figure 3B). Additionally, we detected multipotency of SHED. After induction in osteogenic and adipogenic media, the corresponding staining was performed to detect their differentiation ability; isolated cells had osteogenic and adipogenic differentiation abilities (Figure 3C). Collectively, SHED was isolated successfully.

### Scaffold Materials Have Good Biocompatibility

**FIGURE 4:**
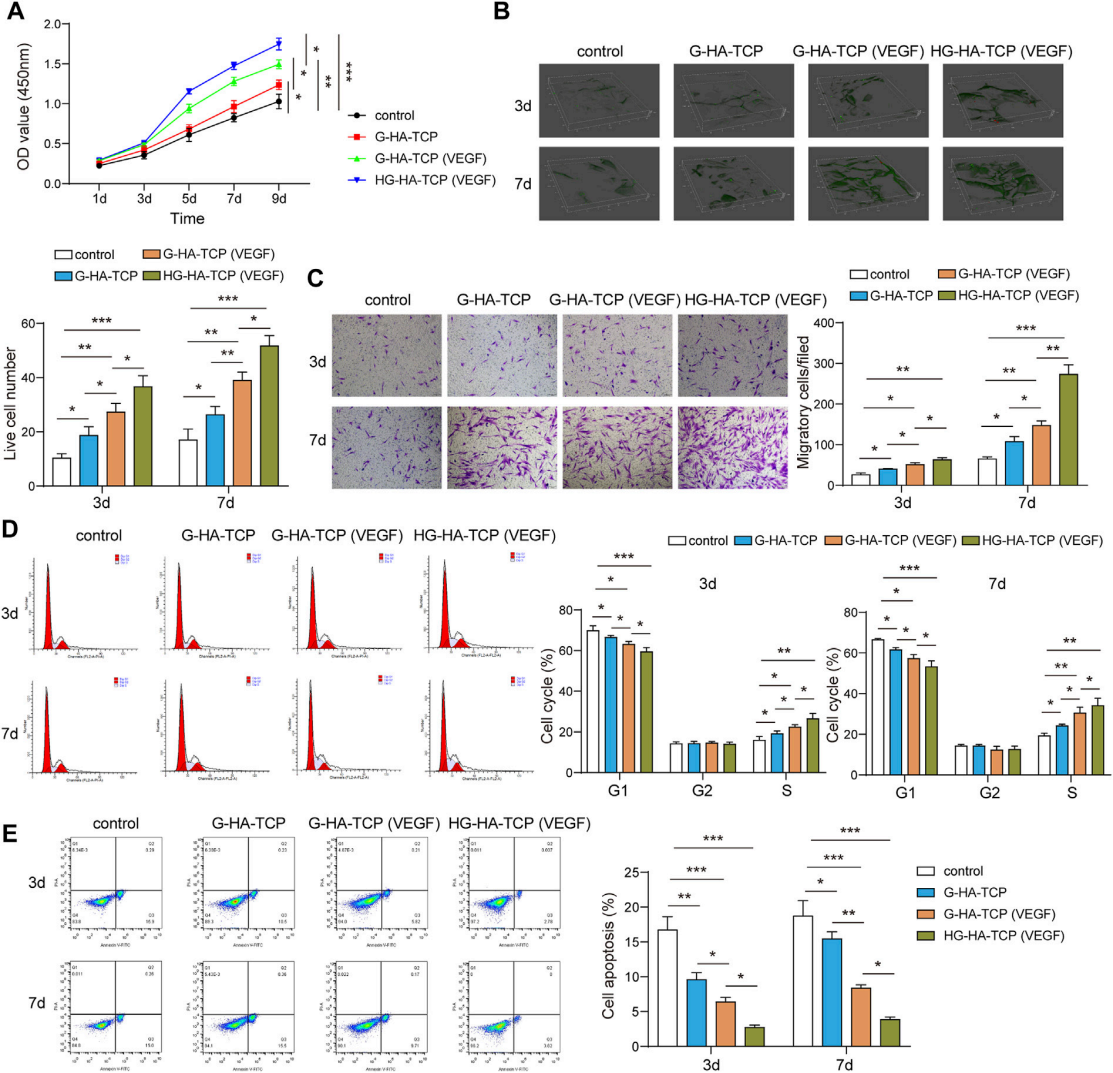
Biocompatibility of scaffold materials. **(A)** Cell proliferation was detected using CCK-8 assay. **(B)** Cell survival was assessed usingLIVE/DEAD assay. **(C)** Transwell assay was performed to measure cell migration. **(D,E)** Flow cytometry was performedfor the detection of cell cycle and apoptosis *n* = 3. *p < 0.05, **p < 0.01, ***p < 0.001.

After co-culturing cells and scaffolds, we first detected cell proliferation usingthe CCK-8 assay. All three scaffolds could promote cell proliferation compared with the scaffold-free control group. G-HA-TCP (VEGF) promoted cell proliferation markedly better than G-HA-TCP but weaker than HG-HA-TCP (VEGF) (Figure 4A). LIVE/DEAD assays revealed that all three scaffold materials increased cell survival rate compared withthe scaffold-free control group. Moreover, cells in HG-HA-TCP (VEGF) group displayed better viability thanother groups (Figure 4B). Additionally, the migrated cells were increased in scaffold groups compared to the scaffold-free control group, and the number of migrated cells in the G-HA-TCP (VEGF) group was more than that in the G-HA-TCP scaffold groupbut less than that in HG-HA-TCP (VEGF) group (Figure 4C). Additionally, the number of cells in the G1 phase of HG-HA-TCP (VEGF) and G-HA-TCP (VEGF) groups was lower than that in the G-HA-TCP scaffold group (Figure 4D). The rate of cell apoptosis in the G-HA-TCP (VEGF) group was lower than that in G-HA- TCP scaffold group but higher than that in HG-HA-TCP (VEGF) group (Figure 4E). The above results revealed that HG-HA-TCP (VEGF) scaffold could promote cell proliferation, adhesion, and migration and had good biocompatibility, which could be applied in bone tissue engineering.

### HG-HA-TCP (VEGF) Promotes Osteogenic Differentiation of SHED

**FIGURE 5:**
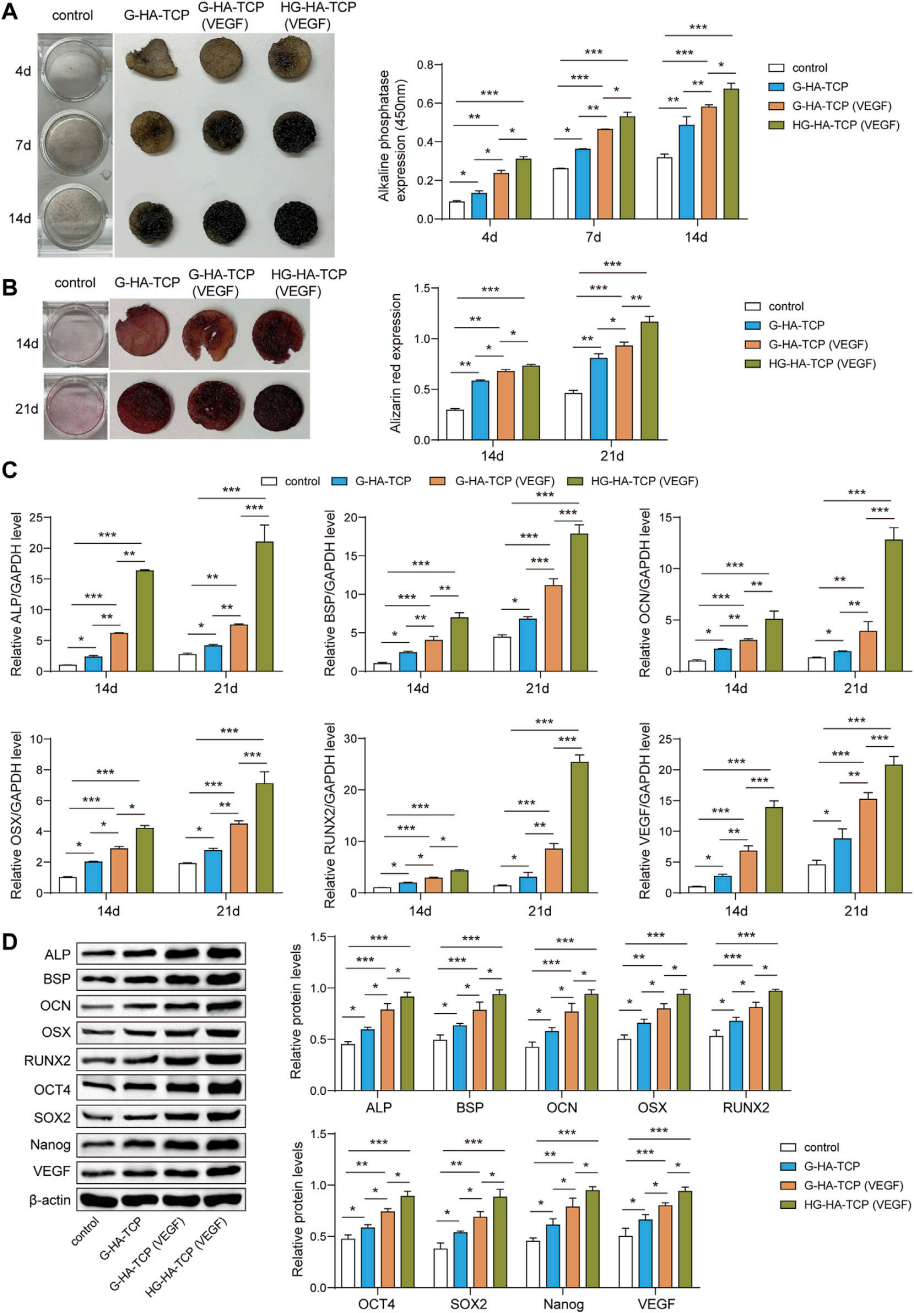
HG-HA-TCP (VEGF) promotes the osteogenic differentiation of SHED. **(A)** ALP staining was performedto detect cell ALP activity. **(B)** Alizarin red S staining was performed to measure cell mineralisation. **(C)** qRT-PCR analysis of mRNA levelsof ALP, BSP, OCN, OSX, RUNX2, and VEGF. **(D)** Western blot analysis of protein expressions of ALP, BSP, OCN, OSX, RUNX2, OCT4, SOX2, Nanog, and VEGF () *n* = 3. *p < 0.05, **p < 0.01, ***p < 0.001.

To further analyse the promoting effect of HG-HA-TCP (VEGF) on osteogenic differentiation of SHED, we first detected ALP activity inthe cells by ALP staining. All three scaffolds improved the osteogenic differentiation of ALP activity compared with the scaffold-free control group. ALP activity in G-HA-TCP (VEGF) group was higher than that in the G-HA-TCP group but lower than that in HG-HA-TCP (VEGF) group (Figure 5A). Results of cell mineralisation detected usingAlizarin red S staining revealed that the content of Alizarin red S in scaffold material groups was increased compared with that in the scaffold-free control group. However, the staining colour of the HG-HA-TCP (VEGF) group was more profoundthan that of the G-HA-TCP (VEGF) group at each time point, and the staining colour of the G-HA-TCP (VEGF) group was deeper than that of G-HA-TCP group (Figure 5B). qRT-PCR revealed that the expressions of BSP, ALP, RUNX2, OSX, OCN, and VEGF were upregulated in the cells of the HG-HA-TCP (VEGF) group, which werebetter than thoseinthe G-HA-TCP (VEGF) and G-HA-TCP groups (Figure 5C). Variations of ALP, BSP, OCN, OSX, RUNX2, OCT4, SOX2, Nanog, and VEGF in scaffold material groups increased dramatically. Moreover, HG-HA-TCP (VEGF) group accounted for the highest expressions in terms of the above factors, followed by G-HA-TCP (VEGF) and G-HA-TCP groups (Figure 5D). The HG-HA-TCP (VEGF) scaffold could promote osteogenic differentiation of SHED, and its effect surpassed that of G-HA-TCP (VEGF) and G-HA-TCP scaffolds.

### HG-HA-TCP (VEGF) Promotes Endothelial Cell Angiogenesis

**FIGURE 6:**
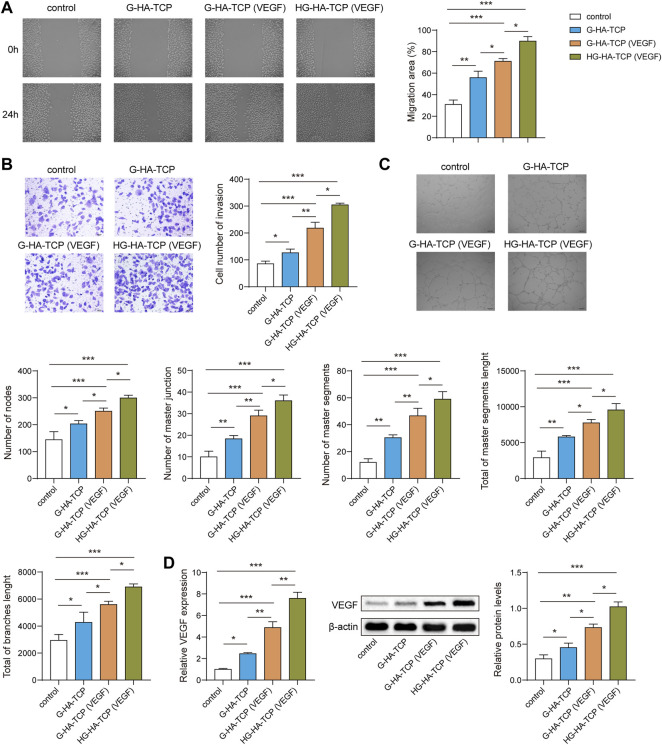
HG-HA-TCP (VEGF) promotes endothelial cell angiogenesis. **(A)** Wound healing assay was performed to detect cell migration. **(B)** Transwell assay was performed for cell invasion measurement. **(C)** Tube formation assay was performed to assess angiogenesis *in vitro*. **(D)** VEGF expression was detected usingqRT-PCR and western blot *n* = 3. *p < 0.05, **p < 0.01, ***p < 0.001.

To analyse the promoting effect of HG-HA-TCP (VEGF) on endothelial cell angiogenesis, we first performeda wound-healing assay to detect cell migration. All three scaffold materials could promote cell migration compared with the scaffold-free control group. The efficacy of the HG-HA-TCP (VEGF) scaffold to promote cell migration was better than that of the G-HA-TCP (VEGF) and G-HA-TCP scaffolds (Figure 6A). Results of cell invasion revealed that all three scaffold materials could promote cell invasion, and the HG-HA-TCP (VEGF) scaffold had a better promoting effect on cell invasion than G-HA- TCP (VEGF) and G-HA-TCP scaffolds (Figure 6B). In tube formation assay, the tube-forming effect of the G-HA-TCP (VEGF) group was slightly better than that of the G-HA-TCP group but was not as good as that of the HG-HA-TCP (VEGF) group (Figure 6C). Compared with the scaffold-free control group, VEGF expression wasincreased markedly in scaffold material groups. Among them, HG-HA-TCP (VEGF) group exhibited the highest amount, and the VEGF level in G-HA-TCP (VEGF) group was higher than that in the G-HA-TCP group (Figure 6D). These results revealed that HG-HA-TCP (VEGF) scaffolds could promote endothelial cell tubule formation, surpassing the efficacy of G-HA-TCP (VEGF) and G-HA-TCP scaffolds.

### HG-HA-TCP (VEGF) Accelerates Bone Regeneration

**FIGURE 7:**
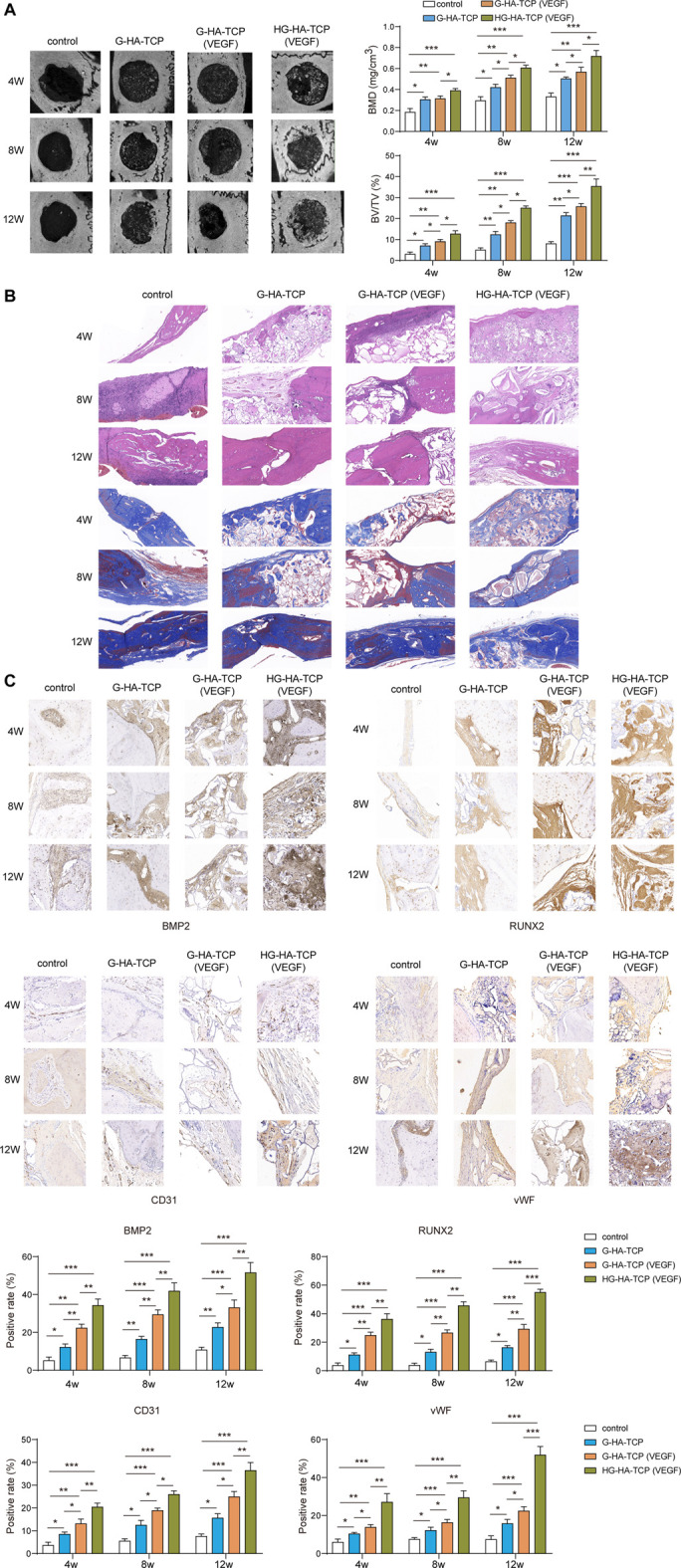
*In vivo* experiments confirmed that HG-HA-TCP (VEGF) could accelerate bone regeneration. **(A)** Micro-CT observation of bone regeneration. **(B)** H&E and Massonstaining for the measurement of bone repair. **(C)** Immunohistochemical detection of BMP2, CD31, RUNX2, and vWF expressions *n* = 6. *p < 0.05, **p < 0.01, ***p < 0.001.

After establishing a mouse model of bone defect, we first used micro-CT to observe the bone regeneration in each group of rats after implanting the scaffolds. Compared with the scaffold-free control group, the new bone in scaffold groups grew concentrically at 4, 8, and 12 weeks, with greater bone mass than that in the blank control group, and BMD and BV/TV increased markedly. In addition, the bone mass, BMD, and BV/TV in HG-HA-TCP (VEGF) group weregreater than thosein G--HA-TCP (VEGF) and G-HA-TCP groups (Figure 7A). Subsequently, H&E and Masson staining was performed for bone repair detection. A small but insignificant amount of new bone formed around the bone defect in a scaffold-free control group, whereasthe three scaffold groups exhibited theformation of a small numberof bone islands, new blood vessels, and fibrous tissue in the scaffold area. Compared with other groups, HG-HA-TCP (VEGF) group exhibited more new bone formation and fibrous tissue (Figure 7B). Further, the expressions of BMP2, CD31, RUNX2, and vWF in the bone tissue were detected using immunohistochemistry, revealing that the above indexes were expressed in a much higher amount in the bone tissue of rats in scaffold groups compared with that in the scaffold-free control group. Among which, HG-HA-TCP (VEGF) scaffold group performed notably better than G-HA-TCP (VEGF) and G-HA-TCP groups in terms of all proteins (Figure 7C). The above results suggested that HG-HA-TCP (VEGF) scaffold accelerated bone regeneration by increasing the osteogenesis-angiogenesis coupling.

## Discussion

Large-scale defects of maxillofacial bone tissue can be caused by oral and maxillofacial trauma, tumours, and congenital deformities, etc., thus seriously affecting the masticatory function, pronunciation, and appearance of patients (Tatullo et al., 2015). Fortunately, defects of bone can be repaired to a favourable extent with tissue engineering technology (El-Rashidy et al., 2017), and ideal bioscaffold material applied in which should possess good biocompatibility, degradability, suitable degradation rate and biomechanical strength, good cell-interface relationship, and structure that is easy to process (Cui et al., 2018; Tian et al., 2021). In this study, it was observed that the porosity, pore size, and mechanical properties of HG-HA-TCP scaffold were similar to those of G-HA-TCP scaffold, which met the needs of bone tissue engineering. Moreover, it was of good biocompatibility and could effectively promote cell adhesion, proliferation, and migration after loading an appropriate amount of VEGF. AndVEGF-loaded HG-HA-TCP scaffolds could promote the expression of osteogenesis-related genes in SHED.

Studies have reported that the shape and size of scaffold pores of bioscaffold materials could affect the migration and growth of seed cells, and ideal pore size should be controlled between 100 and 400 μm (Deschamps et al., 2017). In this study, the pores of HG-HA-TCP scaffold were uniformly distributed; its internal morphology was analogous to that of natural cancellous bone with good integrity of pore wall. The diameter of the pores was 50–200 μm, which was conducive to the exchange of tissue nutrients and metabolic wastes and able to promote the effective growth of new tissue structures. G in scaffolds is a protein of natural origin, which can strengthen the mechanical properties of scaffold materials, make up for their rigidity and is lack of elasticity. The elastic modulus of the scaffoldaresimilar to that of cancellous bone with a relatively flat stress-strain curve, and satisfy the requirements of mechanical properties (Echave et al., 2017; Wiria et al., 2007). As an ideal seed cell in tissue engineering, SHED poses strong proliferation ability, population multiplication rate, and convenience to its source. Itneither causes damage to the donor nor hasimmune rejection or ethical conflicts for autologous use. Combined with biological scaffolds, it can be applied to oral craniomaxillofacial tissue engineering, such as periodontal tissue regeneration and jaw defect repair (Nakamura et al., 2009; Tan et al., 2015). Thus, it was chosen to serve as seed cells in present study and was found that cells could express the mesenchymal stem cell markers CD44, CD90, and CD105, whereas the hematopoietic cell markers CD11b, CD34, CD45, and CD14 were negatively expressed (Figure 2B). Previous studies have also reported that SHED positively expressed the markers CD166, CD146, CD90, CD73, and CD29, but negatively expressed CD45, CD34, and CD14 (Huang et al., 2009; Rodríguez-Lozano et al., 2011), proving that SHED was successfully isolated in our study. Biocompatibility refers to possessing the ability to reproduce tissue, and to bolster cellular viability without causing any toxic action or immune responses from the host (Hussein et al., 2016). And an optimal bone scaffold must be osteoconductive to form new bone by recruiting progenitor cells, biomolecular signalling, and biocompatibility (Kharkar et al., 2013). Compatibility of biomaterials is critical for their biological functions and applications, for the host immune system is the first defender to respond during trauma or biomaterial implantation surgery (Anderson et al., 2008). Application of scaffolds unavoidably induces foreign body reactions, which is likely to cause damage to tissue regeneration and lead to fibrosis and scarring. However, immune responses to tissue damages can also accelerate the healing process to certain extent (Saleh and Bryant, 2018). To further confirm the biocompatibility of the scaffold materials, we conducted related analysis and revealed that HG-HA-TCP (VEGF) scaffold could promote cell proliferation, invasion and migration, and is featured withfavorable biocompatibility.

ALP secreted during osteoblast differentiation is an early bone formation and osteoblast marker, the activity of which can reflect the biological activity of osteoblasts (Siller and Whyte, 2018). And it was suggested from the results that ALP activity of HG-HA-TCP (VEGF) group was markedly greater than that of G-HA-TCP (VEGF) and G-HA-TCP groups on days 4, 7, and 14, suggesting that HG-HA-TCP (VEGF) functioned better in promoting osteogenic differentiation of SHED than that of G-HA-TCP (VEGF) scaffold. Previous study reported that HA can regulate the ALP expression in osteoblasts, that the formation of the mineralised nodule is a sign of late osteogenic differentiation (Yuan et al., 2017), and that Alizarin red S staining mainly uses Alizarin red S to react with calcium nodules deposited in osteogenic-induced extracellular, resulting in dark red-coloured compounds, which can reflect the osteogenic differentiation ability of cells. Results of our study indicated that the exudate of HG-HA-TCP (VEGF) group could significantly promote osteogenic differentiation of SHED and induce its mineralisation. Bone formation is a convoluted course, during the process of which *in vivo*, the expression of bone-related genes has a chronological order and the expression time is strictly controlled, which is essential for bone metabolism, formation, growth, and remodelling (Pan et al., 2017). ALP is produced early in cell development and is readily found on the cell surface and in stromal vesicles of bone and calcified cartilage (Lee et al., 2019). BSP, a marker in the middle and late stages of bone formation, was found in studies to be involved in the attachment and differentiation of fibroblasts, osteoblasts, and osteoclasts, as well as in the process of bone tissue mineralisation; it plays an essential role in periodontal tissue regeneration (Kasugai et al., 1992). RUNX2 can promote the immature differentiation of osteoblasts in early stage and is the earliest and most specific marker in bone formation (Ao et al., 2017). OSX is a negative regulator that is specifically expressed only in osteoblasts. Studies have suggested that overexpression of OSX inhibited the differentiation and maturation of osteoblasts, which revealed that only proper expression of OSX could stimulate bone formation (Yoshida et al., 2012). And results of present study suggested that mRNA levels of RALP, BSP, OCN, OSX, and RUNX2 were markedly elevated on the 14th day in SHED cultured on HG-HA-TCP (VEGF) scaffolds, suggesting that HG-HA-TCP (VEGF) scaffolds could promote SHED osteogenic differentiation at an early stage.

Bone is a highly vascularized connective tissue whose development, maturation, remodelling, and regeneration all depend on tight regulation of vascular supply (Dai and Rabie, 2007). Blood vessels not only supply oxygen or nutrients to the skeletal system and remove metabolites from the bone, but also supply the bone with specific hormones, growth factors, and neurotransmitters secreted by other tissues; they maintain bone cell survival and stimulate their activity (Filipowska et al., 2017). In our study, it was observed that compared with G-HA-TCP scaffold, HG-HA-TCP (VEGF) and G-HA-TCP (VEGF) scaffolds could promote angiogenic gene expression, in which HG-HA-TCP (VEGF) scaffold material had a stronger effect on promoting angiogenesis. This indicated that VEGF could regulate osteogenesis and angiogenesis through a certain signalling pathway. In addition, we observed that HG-HA-TCP (VEGF) scaffold had a stronger effect on the migration and invasion of SHED cells than the G-HA-TCP (VEGF) and G-HA-TCP groups, demonstrating that HG-HA-TCP (VEGF) scaffold material had stronger osteogenic effect than G-HA-TCP (VEGF) and G-HA-TCP groups.

Moreover, previous studies reported that VEGF was one of the most crucial regulators of angiogenesis and was essential for the development and regeneration of the bones (Schipani et al., 2009). VEGF played a dual role in the above processes, activating endothelial cells to accelerate their migration and proliferation and stimulating osteogenesis by regulating osteogenic growth factors (Hu and Olsen, 2016). It not only promoted vascular invasion and cartilage fragmentation tissue recruitment to hypertrophic cartilage but also was required for intramembranous ossification (Percival and Richtsmeier, 2013). Thus, angiogenesis and osteogenesis were tightly linked and must be tightly coupled for the physiological function of bone, as confirmed by our observations.

Previous studies demonstrated that intramembranous bone formation depended on the coupling relationship between angiogenesis and osteogenesis, and VEGF was essential in this coupling because both blood vessel and bone morphogenesis were VEGF-dependent (Tang et al., 2012). Angiogenesis and increased angiogenesis bring in bone-forming progenitor cells and minerals, nutrients, and oxygen required for mineralisation. Moreover, vascular-released osteogenic factors, such as BMP2 accelerate osteoblast differentiation and mineralisation (Matsubara et al., 2012). The disadvantage of *in vitro* studies is that they cannot well-simulate the complex environment *in vivo*, so the osteogenic properties of scaffolds cannot be accurately reflected (Delgado-Ruiz et al., 2015). Therefore, we further evaluated the osteogenic ability of scaffolds through animal experiments. H&E and Masson staining indicated that VEGF-loaded scaffolds had better osteogenic ability, and HG-HA-TCP (VEGF) scaffolds better promoted new bone formation than G-HA-TCP (VEGF). This may be due to the stable loading of VEGF by heparin and its slow release, which expanded the time scale factor for slow-release growth; this, in turn, expanded the range of transportable growth factors for tissue repair and regeneration. A previous study reported that bolus injection of VEGF into the defect without scaffolds did not improve angiogenesis and bone formation, whereas VEGF bolus injection in a hydrogel, released at a relatively slow rate, successfully improved angiogenesis and osteogenesis (Kaigler et al., 2013). Above studies suggested that the release kinetics of VEGF were essential for the effect of biomaterials on bone formation and vascularisation. However, the release kinetics of VEGF in HG-HA-TCP scaffold still need further analysis.

## Conclusion

The porosity, pore size, and mechanical properties of the HG-HA-TCP scaffold could meet the needs of bone tissue engineering with favourable biocompatibility. It could effectively promote cell adhesion, proliferation, and migration after loading an appropriate amount of VEGF. We confirmed that using HG-HA-TCP (VEGF) scaffold material could effectively promote the expression of osteogenesis-related genes in SHED and bone regeneration *in vivo*. The present study provided a theoretical basis for applying this new bone tissue engineering scaffold material in oral implants with insufficient bone mass, which is valuable in clinical practice. However, VEGF has its advantages and disadvantages, and only the appropriate concentration of VEGF can provide the best effect. Further experiments are required to identify a more precise concentration of VEGF, and the related mechanism of how VEGF promotes osteogenesis needs to be further studied.

## Data Availability

The original contributions presented in the study are included in the article/supplementary material, further inquiries can be directed to the corresponding authors.

## Abbreviations

ALP, alkaline phosphatase; BMD, bone mineral density; BSP, bone salivary protein; BV/TV, bone volume/total volume; CCK-8, cell counting kit-8; H&E, haematoxylin eosin; GAPDH, glyceraldehyde-3-phosphate dehydrogenase; HG-HA-TCP, heparinised gelatin-hydroxyapatite-tricalcium phosphate; OCN, osteogenesis-related gene osteocalcin; OSX, osterix; qRT-PCR, quantitative real time polymerase chain reaction; RUNX2, runt-related transcription factor 2; SHED, stem cells fromhuman exfoliated deciduous teeth; VEGF, vascular endothelial growth factor.
